# SNHG10 Is a Prognostic Biomarker Correlated With Immune Infiltrates in Prostate Cancer

**DOI:** 10.3389/fcell.2021.731042

**Published:** 2021-10-05

**Authors:** Qiang Chen, Xiaorong Yang, Binbin Gong, Wenjie Xie, Ming Ma, Shengqiang Fu, Siyuan Wang, Yutang Liu, Zhicheng Zhang, Ting Sun, Zhilong Li

**Affiliations:** Department of Urology, The First Affiliated Hospital of Nanchang University, Nanchang, China

**Keywords:** SNHG10, prostate cancer, prognosis, biomarker, proliferation

## Abstract

SNHG10 is a long non-coding RNA (lncRNA) found to be overexpressed in multiple human cancers including prostate cancer (PC). However, the underlying mechanisms of SNHG10 driving the progression of PC remains unclear. In this study, we investigated the role of SNHG10 in PC and found that SNHG10 expression was significantly increased in datasets extracted from The Cancer Genome Atlas. Increased expression of SNHG10 was related to advanced clinical parameters. Receiver operating curve analysis revealed the significant diagnostic ability of SNHG10 (AUC = 0.805). In addition, immune infiltration analysis, and GSEA showed that SNHG10 expression was correlated with oxidative phosphorylation and immune infiltrated cells. Finally, we determined that SNHG10 regulated cell proliferation, migration, and invasion of PC *in vitro*. In conclusion, our data demonstrated that SNHG10 was correlated with progression and immune infiltration, and could serve as a prognostic biomarker for PC.

## Introduction

Prostate cancer (PC) is the most frequently diagnosed malignancy among men worldwide accounting for approximately 21% of new cancer cases in 2020 (Siegel et al., 2020). With the development of magnetic resonance imaging (MRI) and prostate specific antigen (PSA) screening, detection of PC has increased (Hayes and Barry, 2014; Rastinehad et al., 2014). Although androgen deprivation therapy (ADT) has improved the prognosis of patients with localized PC, progression to castration-resistant PC is inevitable. Therefore, it is critical to identify significant biomarkers for diagnosing the occurrence and progression of PC.

Long noncoding RNAs (lncRNAs) belong to a subset of non-coding RNA transcripts of over 200 nucleotides in length (Shi et al., 2013; Hua et al., 2019; Goodall and Wickramasinghe, 2021). Recent studies have indicated that lncRNAs might play a crucial role in the progression of different cancers (De Troyer et al., 2020; Luo et al., 2020; Statello et al., 2021). For example, the lncRNA NEAT1 promotes PC metastasis to the bone (Wen et al., 2020). LncRNA PVT1 plays a pivotal role in PC progression by inhibiting KIF23 expression via enriched miR-15a-5p levels (Wu et al., 2020). Small nucleolar RNA host gene 10 (SNHG10) has been reported to be a cancer-promoting gene in various human cancers. For instance, SNHG10 promotes cell proliferation and invasion in osteosarcoma (Zhu et al., 2020). Recently, SNHG10 has been identified as having a tumor facilitator role in hepatocellular carcinoma and gastric carcinoma (Lan et al., 2019; Zhang et al., 2020). However, the clinical value of SNHG10 in PC has not been explored. Hence, this study aimed to investigate the role of SNHG10 in the progression of PC.

In this study, we compared the expression of SNHG10 between PC tissues and normal samples, and investigated the correlation between SNHG10 expression and clinical parameters of PC. In addition, we explored the prognostic value and clinical significance of SNHG10 in PC. Meanwhile, the correlation between SNHG10 expression and immune infiltration was analyzed to explore the potential mechanisms involved in SNHG10 modulation in the carcinogenesis of PC. Finally, the biological role of SNHG10 was identified in PC. In summary, we demonstrated that the potential role of SNHG10 in regulating tumor progression and its potential application in the diagnosis and prognostic evaluation in PC.

## Materials and Methods

### Data Source

The gene expression profiles of PC patients were downloaded from The Cancer Genome Atlas (TCGA) database^1^, as well as the corresponding clinical and DNA methylation information. This included 499 PC samples and 52 adjacent normal tissues which were retrospectively studied. For validation, additional PRAD cohorts of 293 and 248 patients were obtained with the accession number GSE70770 and GSE116918 from the GEO database^2^. Gene expression data and clinical data were downloaded.

### Construction and Validation of the Nomogram

Firstly, patients that lack information of T stage, N stage, PSA, Gleason score, residual tumor, SNHG10 expression, and survival status were excluded from following analysis. Then, 288 prostate cancer samples were screened from the TCGA data. These samples were randomly classified into the training cohort (*n* = 144) and the test cohort (*n* = 144). Subsequently, we developed the nomogram in the training cohort using cox regression analysis, and validated in the test cohort. R package “rms” was used to establish the nomogram for predict the probability of 3-year and 5-year survival. The ROC curve for nomogram was performed via “timeROC” R package. The calibration curve was conducted via “calibrate” function of “rms” R package. We performed the DCA analysis of survival outcome by “ggDCA” R package.

### Gene Set Enrichment Analysis

The Gene set enrichment analysis (GSEA) was downloaded from the GSEA website^3^. A customized Perl script^4^ was used to perform GSEA between high-SNHG10 and low-SNHG10 groups. According to the default statistical methods, an adjusted *P*-value < 0.05 was considered significant.

### Immune Infiltration Analysis by Single Sample Gene Set Enrichment Analysis

Single sample GSEA (ssGSEA) using the R package “GSVA” was applied to perform the immune infiltration analysis of PC. The infiltration levels of 24 immune cell types were determined based on gene expression profiles in the literature (Bindea et al., 2013; Hänzelmann et al., 2013). The correlation between SNHG10 and the infiltration levels of 24 immune cells was evaluated by Spearman’s test and the Wilcoxon rank sum test. In addition, TIMER website^5^ was used to further investigate the association between immune infiltration and the expression of SNHG10, which is a comprehensive resource for systematical analysis of immune infiltrates across diverse cancer types.

### Cell Culture and siRNA Transfection

Human PC cell lines (VCaP, LNCaP, 22RV1, PC3, and DU145) and the human normal human prostate epithelial cell line (RWPE-1) were purchased from the American Type Culture Collection (ATCC^6^, United States). LNCaP, 22RV1, and DU145 cells were cultured in RPMI-1640 (Gibco, United States). PC3 cells were cultured in F12K medium (Gibco, Australia). VCaP and RWPE-1 cells were cultured in DMEM (Gibco, United States). All cells were cultured with 10% fetal bovine serum (Gibco, Australia) at 37°C in a 5% CO_2_ atmosphere. SNHG10 siRNAs were synthesized by Hanbio (Shanghai, China). The sequences of the siRNAs were as follows: si-SNHG10: 5′-CAACCGCUUUGUUAGUUAATT-3′; si-NC: 5′-UUCUCCGAACGUGUCACGUTT-3′. For the silencing of SNHG10 in the DU145 and 22RV1 cells, the cells were transfected with 50 nM small interfering RNA targeting si-SNHG10 or negative control si-NC according to the manufacturer’s instructions of Lipofectamine 2000 (Invitrogen). Scrambled siRNAs were used as negative control.

### RNA Extraction and Quantitative Real-Time PCR

Total RNA was isolated and reversely transcribed into cDNA using Trizol reagent (Thermo Fisher Scientific, Inc.) and TaKaRa Prime Script RT Reagent Kit (TaKaRa, China) based on the manufacturer’s protocols. RT-qPCR was performed using SYBR Premix Ex Taq (TaKaRa, China) according to standard methodology. The relative expression levels were calculated using the 2^–ΔΔCt^ method relative to GAPDH. The PCR primers used were as follows: GAPDH: 5′-AAAAGCATCACCCGGAGGAGAA-3′ (forward) and 5′-AAGGAAATGAATGGGCAGCCG-3′ (reverse); SNHG10: 5′-GTTGGTCTCTTGGGAGGTAG-3′ (forward) and 5′-CGCC ACGACGAACTGCATGC-3′ (reverse). All experiments were performed in triplicate.

### Cell Proliferation

Cell Counting Kit 8 (CCK-8) and colony formation assays were used to measure cell proliferation. For CCK-8 assays, PC cells were seeded into a 96-well plate (6000 cells per well). CCK-8 reagent (Hanbio, China) was added into wells at 24, 48, and 72 h. After the cells were incubated at 37°C for 2 h, the optical density (OD) was measured at 450 nm using a microplate reader. For colony formation, 2000 cells per well were seeded in 6-well plates in 3 mL medium per well. After approximately 2 weeks, cell colonies were fixed with 4% paraformaldehyde for 15 min. Then, the colonies were stained with 0.2% crystal violet (Solarbio, China) and counted using ImageJ software. Each experiment was repeated three times.

### Transwell Assay

Cell migration assays were performed using 8-μm pore size chambers coated without Matrigel gel. A total of 5 x 10^4^ transfected cells were seeded into the upper chamber with serum-free medium and 20% FBS medium was added into the lower well. After incubation for 1 day, the cells were stained and observed under an optical microscope. Three random fields were analyzed for each sample. Cell invasion assays were performed using chambers with Matrigel gel. Other procedures were the same as above. All the assays were conducted three times independently.

### Statistical Analysis

For the datasets from TCGA database, statistical analyses were performed using R (v.3.6.3). The Wilcoxon rank sum test, Chi-square test, and Fisher exact test were used to estimate the association between SNHG10 and clinical pathologic characteristics. The Kaplan-Meier method was used to calculated PC patient survival rates. Univariate and multivariate cox analysis were performed to assess the relationship between clinical features and progression-free survival (PFS). For the data regarding the function of SNHG10, GraphPad Prism 7.01 was used for statistical analyses. The Student’s t-test evaluated the statistical significance between groups. The data was shown as mean ± SD from at least three independent experiments and *P* < 0.05 was considered statistically significant.

## Results

### SNHG10 Was Over-Expressed in Prostate Cancer

**FIGURE 1:**
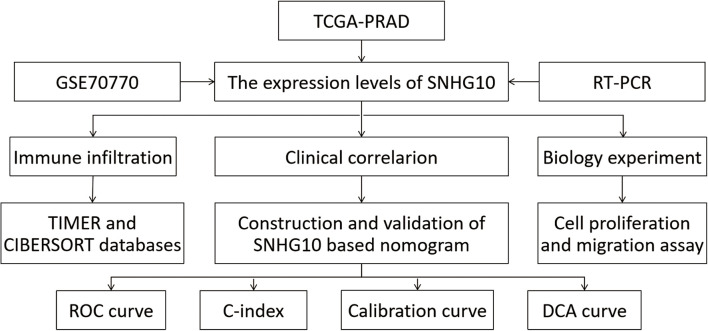
The workflow of this study.

**FIGURE 2:**
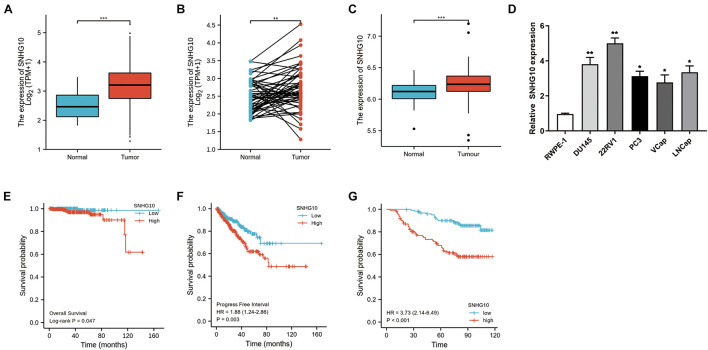
SNHG10 exhibits prognostic value in PC. **(A)** Comparison of SNHG10 expression between 499 PC tissues and 52 adjacent prostate tissues from TCGA-PRAD. **(B)** Paired comparison of SNHG10 expression between 52 PC tissues and 52 matching adjacent prostate tissues from TCGA-PRAD. **(C)** SNHG10 expression between 219 PC tissues and 74 normal tissues in GSE70770. **(D)** SNHG10 expression was significantly higher in PC cell lines (VCaP, LNCaP, 22RV1, PC3, and DU145) compared with the prostate cell line (RWPE-1). Kaplan-Meier survival analysis was performed to determine differences in **(E)** OS and **(F)** PFS between the high-SNHG10 and low-SNHG10 in TCGA-PRAD. **(G)** Kaplan-Meier analysis in GSE70770. *P < 0.05, **P < 0.01, and ***P < 0.001.

The workflow of this study is presented in Figure 1. To compare the expression of SNHG10 in PC and normal samples, we analyzed the expression of SNHG10 in 499 tumor tissues and 52 normal prostate tissues of TCGA data, and found that SNHG10 was over-expressed in PC tissues (*P* < 0.001) (Figure 2A). There were 52 pairs of cancer samples and matched adjacent normal samples in TCGA data. The expression of SNHG10 was also higher in cancer samples than matched adjacent normal samples (*P* < 0.01) (Figure 2B). In addition, we compared the expression of SNHG10 between normal and prostate cancer in GSE70770, and found that SNHG10 was over-expressed in cancer tissues (*P* < 0.001) (Figure 2C). Meanwhile, SNHG10 expression was significantly higher in PC cell lines (DU145, 22RV1, PC3, VCaP, and LNCaP) than the prostate cell line (RWPE-1) (Figure 2D). Additionally, Kaplan-Meier survival analysis was used to investigate the relationship of SNHG10 expression and overall survival (OS) or progression-free survival (PFS) in the PC patients of TCGA data. SNHG10 expression has great significance in predicting OS (*P* < 0.05) (Figure 2E). As shown in Figure 2F, SNHG10 expression was significantly associated with poor PFS of PC patients (*P* = 0.003). Additionally, the concordance-index (C-index) was calculated to evaluate SNGH10 expression in predicting the OS and PFS. The C-index for SNGH10 in predicting the OS was 0.619. The performance of SNHG10 for predicting the PFS showed a better prognostic power with a C-index of 0.727. Then, patients with high SNHG10 expression had a worse PFS compared with low expression group in GSE70770 (*P* < 0.001) (Figure 2G).

### Overexpression of SNHG10 Was Associated With Poor Clinical Parameters in Prostate Cancer

**FIGURE 3:**
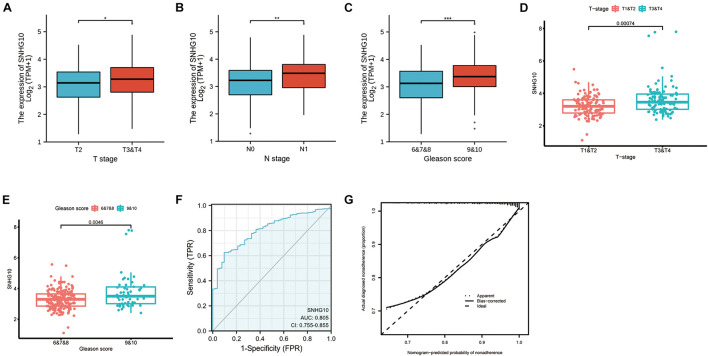
SNHG10 was associated with clinical parameters in PC. SNHG10 expression levels among PC patients with different **(A)** T stage, **(B)** N stage, and **(C)** Gleason score in TCGA-PRAD. SNHG10 expression levels with different **(D)** T stage, **(E)** Gleason score in GSE116918. **(F)** Diagnostic efficacy of the ROC curve of SNHG10 expression in TCGA-PRAD. **(G)** Calibration curves of SNGH10 expression in TCGA-PRAD. ROC, receiver operating characteristic; AUC, area under the ROC curve; CI, confidence interval. *P < 0.05, **P < 0.01, and ***P < 0.001.

Overall, 499 PC patients with clinical parameters were classified into two subgroups according to the mean value of relative SNHG10 expression. We then analyzed the relationships between SNHG10 and clinical parameters, including T stage, N stage, M stage, Gleason score, primary therapy outcome, residual tumor, zone of origin, prostate specific antigen (PSA), age, and race. SNHG10 expression was significantly associated with T stage (*P* = 0.013), N stage (*P* = 0.005), Gleason score (*P* < 0.001), primary therapy outcome (*P* < 0.001), and race (*P* = 0.008), while patients of different ages, M stage, residual tumor, zone of origin, and PSA shown no significant difference (Table 1). To visualize and clarify these relationships, we divided patients into subgroups (T2 vs. T3+T4, N0 vs. N1, Gleason score 6+7+8 vs. Gleason score 9+10). As shown in Figures 3A–C, advanced T stage, lymph node, and Gleason score patients exhibited higher SNHG10 expression levels. Moreover, in the GSE116918, SNHG10 expression was significantly correlated with advanced T stage and Gleason score (Figures 3D,E). Then, ROC analysis showed that the SNHG10 could be used to differentiate PC patients from normal control with a cut-off of 2.31 which resulted in 59.9% for sensitivity and 90.2% for specificity (AUC = 0.805, C-index = 0.806, Youden index = 0.501) (Figure 3F). Besides, calibration curve of SNGH10 were coincident with the reference line, which indicated a high degree of credibility (Figure 3G).

**TABLE 1:** Correlation between SNHG10 expression and clinical parameters in PC.

**Clinical parameters**	**Levels**	**Low expression of SNHGI0**	**High expression of SNHGI0**	***P*-value**
n		249	250	
T stage, n (%)	T2	101(20.5%)	88(17.9%)	**0.013**
	T3/T4	144(29.3%)	159(32.3%)	
N stage, n (%)	N0	173(40.6%)	174(40.8%)	**0.005**
	N1	30(7%)	49(11.5%)	
M stage, n (%)	M0	226(49.3%)	229(50%)	1.000
	M1	1(0.2%)	2(0.4%)	
Gleason score, n (%)	6	26(5.2%)	20(4%)	<**0.001**
	7	147(29.5%)	100(20%)	
	8	21(4.2%)	43(8.6%)	
	9	52(10.4%)	86(17.2%)	
	10	3(0.6%)	1(0.2%)	
Primary therapy outcome, n (%)	PD	17(3.9%)	11(2.5%)	**<0.001**
	SD	8(1.8%)	21(4.8%)	
	PR	12(2.7%)	28(6.4%)	
	CR	188(42.9%)	153(34.9%)	
Residual tumor, n (%)	R0	168(35.9%)	147(31.4%)	0.112
	R1	66(14.1%)	82(17.5%)	
	R2	4(0.9%)	1(0.2%)	
Zone of origin, n (%)	Central Zone	2(0.7%)	2(0.7%)	0.541
	Overlapping/Multiple Zones	48(17.5%)	78(28.4%)	
	Peripheral Zone	43(15.6%)	94(34.2%)	
	Transition Zone	2(0.7%)	6(2.2%)	
PSA (ng/ml), n (%)	<4	211(47.7%)	204(46.2%)	0.942
	≥4	13(2.9%)	14(3.2%)	
Age, n (%)	≤60	120(24%)	104(20.8%)	0.164
	> 60	129(25.9%)	146(29.3%)	
Race, n (%)	Asian	4(0.8%)	8(1.7%)	**0.008**
	Black or African American	19(3.9%)	38(7.9%)	
	White	222(45.9%)	193(39.9%)	
Age, meidan (IQR)		61(56,66)	62(56,66)	0.430
PSA (ng/ml), meidan		0.1(0.03,0.1)	0.1(0.02,0.2)	0.916

*SD, stable disease; PD, progressive disease; PR, partial response; CR, complete response; PSA, prostate specific antigen. Bold values represent the statistically significant p-values (P < 0.05).*

### Overexpression of SNHG10 Expression Was Correlated With Poor Prognosis in Numerous Cancers

**FIGURE 4:**
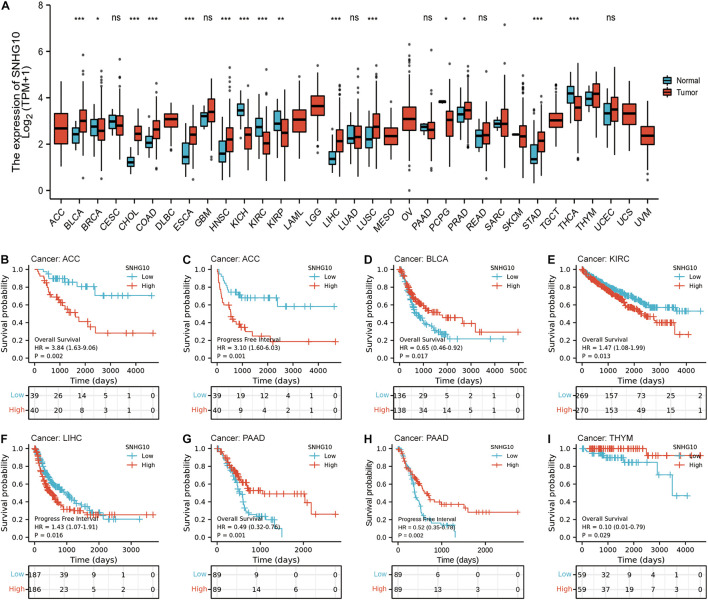
SNHG10 expression and prognosis in patients with other cancer types. **(A)** Differences in expression of SNHG10 in normal and tumor tissues of TCGA. **(B–I)** Kaplan-Meier curves were drawn to evaluate the prognostic value of SNHG10 in OS or PFS of other cancers. *P < 0.05, **P < 0.01, and ***P < 0.001.

TCGA database was used to identify the aberrant expression of SNHG10 across multiple cancers. As shown in the Figure 4A, SNHG10 expression was higher in multiple cancer types compared to the normal samples. In addition, SNHG10 expression and patient survival was further investigated in various cancer types to expand our analysis to a pan-cancer level. There are significant associations between SNHG10 expression and OS or PFS in adrenocortical carcinoma (ACC), bladder urothelial carcinoma (BLCA), kidney renal clear cell carcinoma (KIRC), liver hepatocellular carcinoma (LIHC), pancreatic adenocarcinoma (PAAD), and thymoma (THYM) (Figures 4B–I).

### Cox Univariate and Multivariate Analysis of Prognostic Factors in Prostate Cancer

As shown in the Table 2, patients having complete clinical data were included in further Cox regression analysis. In the Cox univariate regression analysis, high expression of SNHG10, T stage, N stage, Gleason score, primary therapy outcome, residual tumor, and PSA were associated with PFS in PC patients. Multivariate Cox analysis further indicated that SNHG10 (*P* < 0.05) was an independent prognostic factor for PFS in PC patients, along with Gleason score and primary therapy outcome.

**TABLE 2:** Univariate and multivariate regression analyses of PC.

	**Total (N)**	**Univariate analysis**		**Multivariate analysis**	
**Variables**		**Hazard ratio (95% Cl)**	***P* value**	**Hazard ratio (95% Cl)**	***P* value**
T stage (T3&T4 vs. T2)	492	3.785 (2.140-6.693)	**<0.001**	1.512 (0.722-3.165)	0.273
N stage (N1 vs. N0)	426	1.946 (1.202-3.150)	**0.007**	0.803 (0.455-1.417)	0.449
M stage (M1 vs. M0)	458	3.566 (0.494-25.753)	0.208		
Gleason score (9&10 vs. 6&7&8)	499	4.590 (3.038-6.934)	**<0.001**	2.385 (1.374-4.140)	**0.002**
Primary therapy					
outcome (CR vs.	438	0.151 (0.099-0.231)	**<0.001**	0.283 (0.159-0.502)	**<0.001**
PD&SD&PR)					
Residual tumor (R1&R2 vs. R0)	468	2.365 (1.566-3.570)	**<0.001**	0.961 (0.566-1.632)	0.883
PSA (ng/ml) (≥4 vs. <4)	442	4.196 (2.095-8.405)	**<0.001**	1.711 (0.764-3.829)	0.191
Age (>60 vs. ≤60)	499	1.302 (0.863-1.963)	0.208		
Race					
(Black or African American vs.	484	0.579 (0.290-1.154)	0.120		
White&Asian)					
Zone of origin					
(Peripheral Zone	263	0.799	0 363		
vs. Overlapping/Multiple Zones)		(0.492-1.296)			
SNHG10 (High vs. Low)	499	1.874 (1.232-2.849)	**0.003**	1.651 (1.012-2.694)	**0.045**

*SD, stable disease; PD, progressive disease; PR, partial response; CR, complete response; PSA, prostate specific antigen. Bold values represent the statistically significant p-values (P < 0.05).*

### Construction and Validation of SNHG10 Based Nomogram

**FIGURE 5:**
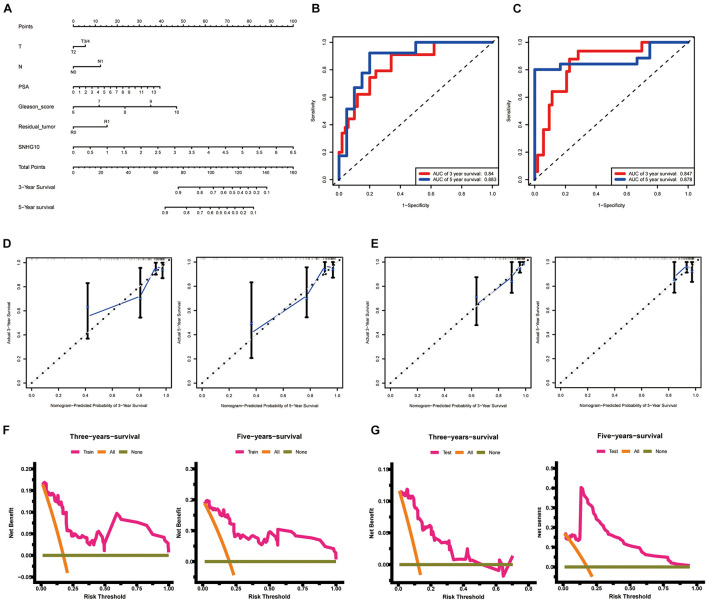
Construction and validation of the nomogram. **(A)** The nomogram for predicting 3-year and 5-year PFS of PC patients. The nomogram AUC of values for 3-year and 5-year PFS **(B)** in the training cohort and **(C)** the test cohort. The calibration plots **(D)** in the training cohorts and **(E)** the test cohorts. DCA curves **(F)** in the training cohorts and **(G)** the test cohorts. ROC, receiver operating characteristic; AUC, area under the ROC curve; DCA, decision curves analysis.

To allow clinical application of our findings, we construct the nomogram by TCGA data. The nomogram for predicting 3-year and 5-year PFS of PC was showed in Figure 5A. ROC curve, calibration, and discrimination were employed to evaluate the performance of the model. The C-index of the nomogram were 0.848 for the training cohort and 0.825 for the test cohort. The discriminative ability of the nomogram was measured using the 3-year and 5-year survival AUC values from time-dependent ROC curve. In the training cohort, the nomogram AUC values for 3-year and 5-year PFS were 0.84 and 0.883, respectively (Figure 5B). In addition, in the test cohort, the nomogram AUC values for 3-year and 5-year PFS were 0.847 and 0.878, respectively (Figure 5C). Moreover, the calibration plots in the training cohorts and test cohorts demonstrated that the nomogram-based predictive results were mostly consistent with the actual prognosis results (Figures 5D,E). DCA plots showed that our nomogram had great net benefits for predicting 3-year and 5-year PFS of patients both in the training cohorts and test cohorts (Figures 5F,G), demonstrating its application in guiding clinical decision for PC patients.

### Correlation Between SNHG10 Expression and Immune Infiltration

**FIGURE 6:**
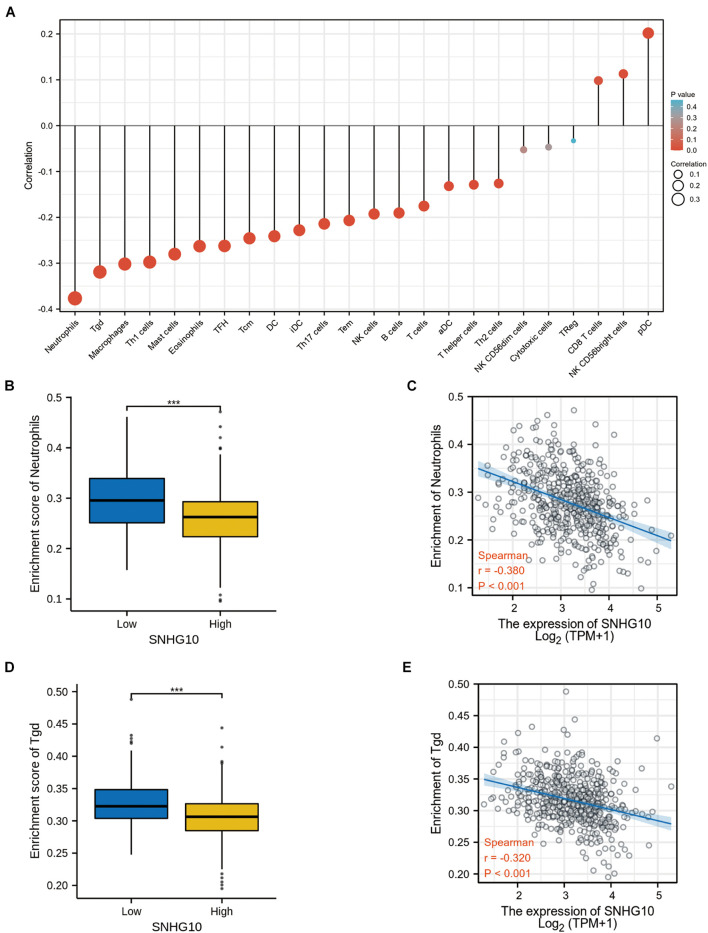
SNHG10 expression associates with immune infiltration in the tumor microenvironment. **(A)** The forest plot shows the correlation between SNHG10 expression and 24 immune cell types. **(B)** Differences in neutrophils cell infiltration between SNHG10 low and high expression groups. **(C)** The correlation between SNHG10 expression and proportion of neutrophils. **(D)** Differences in Tγδ infiltration between SNHG10 low and high expression groups. **(E)** Correlation between SNHG10 expression and Tγδ. Tγδ, T gamma delta. ***P < 0.001.

**FIGURE 7:**
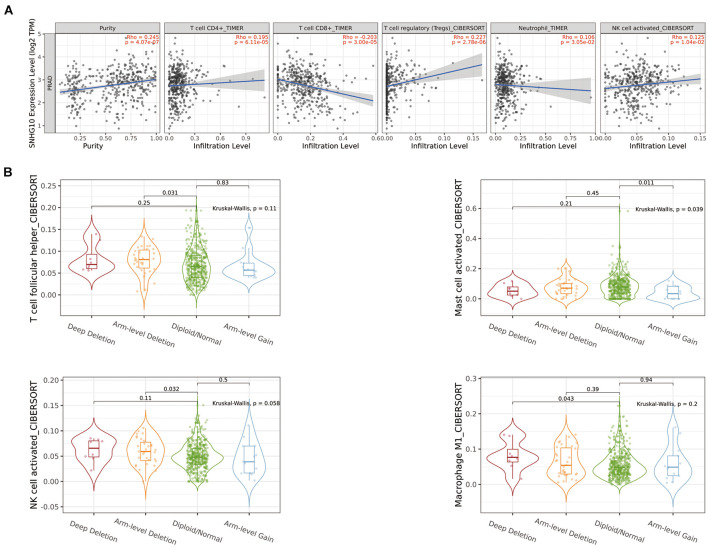
The relationship between SNHG10 expression and tumor-infiltrated immune cells. **(A)** Significant correlation between SNHG10 expression and different type of immune cells. **(B)** Different mutational forms of SNHG10 were associated with immune infiltrates of four leukocytes.

Using the ssGSEA method, we analyzed the relationship between SNHG10 expression and immune infiltration. These results suggested that SNHG10 expression was negatively associated with infiltration levels of neutrophils and T gamma delta (γδT) (*P* < 0.001) (Figure 6), and was positively correlated with that of natural killer (NK) CD56bright cells and plasmacytoid dendritic cells (pDC) (*P* < 0.001) (Supplementary Figure 1). In addition, the tool of TIMER was also used to identify the relationship between SNHG10 expression and tumor-infiltrated immune cells. There was a significant correlation between SNHG10 expression and different type of immune cells, including CD4+ T cells (*P* = 6.11e–5, cor = 0.195), CD8+ T cells (*P* = 3e−5, cor = −0.203), T cells regulatory (*P* = 2.78e−6, cor = 0.227), Neutrophils (*P* = 3.05e–2, cor = 0.106), and NK cells activated (*P* = 1.04e–2, cor = 0.125) (Figure 7A). Besides, different mutational forms of SNHG10 were associated with immune infiltrates of four leukocytes, which also revealed its influence on immune microenvironment (Figure 7B).

### SNHG10-Related Signaling Pathways Based on Gene Set Enrichment Analysis

**FIGURE 8:**
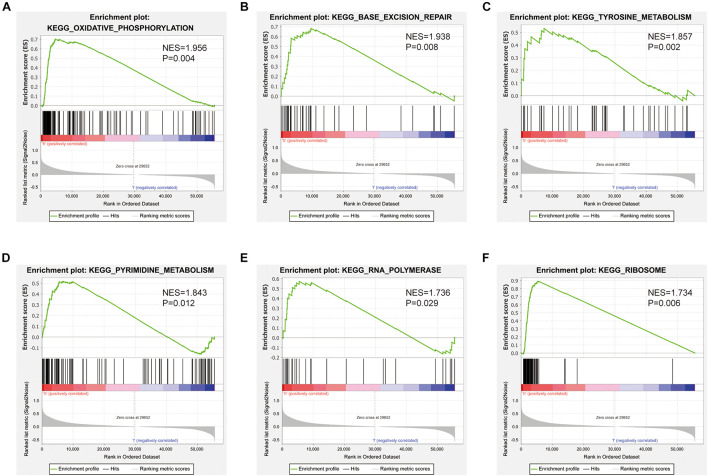
Pathways involved in the pathogenesis of SNHG10 from the GSEA. Enrichment plots comparing SNHG10 expression in terms of **(A)** oxidative phosphorylation, **(B)** base excision repair, **(C)** tyrosine metabolism, **(D)** pyrimidine metabolism, **(E)** RNA polymerase, and **(F)** ribosome.

Gene set enrichment analysis was used to identify signaling pathways that were differentially activated between low and high SNHG10 expression groups. As shown in Figures 8A–F, there were six significant Kyoto Encyclopedia of Genes and Genomes pathways related with the high SNHG10 expression phenotype: oxidative phosphorylation, base excision repair, tyrosine metabolism, pyrimidine metabolism, RNA polymerase, and ribosome.

### SNHG10 Promoted Cell Proliferation, Migration, and Invasion in Prostate Cancer

**FIGURE 9:**
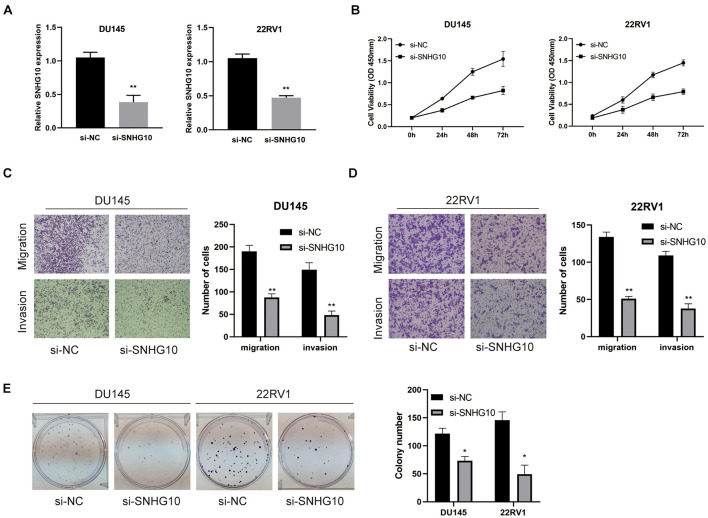
The biological role of SNHG10 on PC cell proliferation, migration, and invasion. **(A)** SNHG10 expression was verified after transfection in DU145 and 22RV1. **(B)** CCK8 assay showing attenuated DU145 and 22RV1 cell growth after knocking down SNHG10 expression. **(C,D)** Migration and invasion abilities of DU145 and 22RV1 were weakened after knocking down SNHG10. **(E)** Knockdown of SNHG10 inhibited colony formation and cell growth of DU145 and 22RV1. The data represent the mean ± SD from three independent experiments. *P < 0.05 and **P < 0.01.

The above studies indicated that SNHG10 expression was distinctly up-regulated in PC tissues, and SNHG10 might influence the progression in PC. To further investigate the biological role of SNHG10 in PC, specific siRNA was used to construct DU145 and 22RV1 cells with stable knockdown of SNHG10 expression (Figure 9A). Our results demonstrated that, in DU145 and 22RV1 cells, cell growth was dramatically inhibited in SNHG10 knockdown cells compared with the control groups (Figure 9B). Next, Transwell assays showed that SNHG10 knockdown suppressed the migratory and invasive abilities of DU145 and 22RV1 cells (Figures 9C,D). SNHG10 inhibition also decreased tumor cell colony formation (Figure 9E). These results demonstrated that SNHG10 was involved in PC progression by inhibiting cell growth, migration, and invasion.

## Discussion

Long non-coding RNAs exert diverse regulatory roles in physiological and pathological processes, including interaction with mRNAs, proteins and miRNAs, to regulate gene expression and induce chromatin remodeling (Kopp and Mendell, 2018; Chi et al., 2019). Previous studies have revealed that lncRNAs were related to various malignant biological behaviors of cancer, such as cell proliferation, apoptosis, migration, and invasion (Sanchez Calle et al., 2018; Yang et al., 2020). It has also been reported that lncAMPC serves as prognostic biomarker in PC (Zhang et al., 2020). Combining bioinformatics analyses and biological function validation, our study provides evidence that SNHG10 was overexpressed in PC, which was also associated with poor prognosis. Aberrant expression of SNHG10 has been elucidated in various human cancers. For example, SNHG10 exerted oncogenic functions in glioma by sponging miR-532-3p and enhancing FBXL19 expression (Jin et al., 2020). In addition, SNHG10 has reported to promote cell proliferation in osteosarcoma via increasing glucose uptake and miR-218 gene methylation (He et al., 2020). SNHG10 facilitates gastric cancer cell proliferation and migration by targeting the miR-495/CTNNB1 axis and activating the WNT pathway (Yuan et al., 2020). All these studies indicated that SNHG10 might exert a pivotal function in human cancers. In our study, we observed increased SNHG10 in PC, which was also associated with poor prognosis. Similarly, high expression of SNHG10 was related to overall survival in hepatocellular carcinoma and non-small cell lung cancer (Lan et al., 2019; Liang et al., 2020).

In this study, we investigated the underlying mechanisms through which SNHG10 influenced the progression of PC. GSEA demonstrated that SNHG10 was significantly associated with oxidative phosphorylation, tyrosine metabolism, and pyrimidine metabolism, which indicated that SNHG10 might have a crucial role in cell metabolism. In addition to the above, we also explored the relationship between the expression of SNHG10 and diverse immune infiltration levels in PC. In our study, we found a moderate correlation between SNHG10 expression and the infiltration of neutrophils, γδT cells, and macrophages in PC. These results could indicate that SNHG10 may inhibit the function of neutrophils and macrophages, and may promote the function of plasmacytoid dendritic cells and NK CD56 bright cells, and thus exert a pro-carcinogenic role in PC. *In vitro*, knockdown of SNHG10 in 22RV1 and DU145 cells impaired cell proliferation, migration, and invasion. Based on these findings, we proposed that SNHG10 exerts an essential function in regulating pathologic progression of PC.

To the best of our knowledge, this is the first study to explore the relationship between SNHG10 and PC. However, there are some limitations in our research. First, our study was based on expression data extracted from TCGA, but may be more convincing if supported by validation studies using other public datasets. Second, the our study was based on a retrospective analysis, and our conclusions should be investigated by a prospective clinical study. Finally, the biological functions of SNHG10 need to be further explored.

## Conclusion

Our study found that SNHG10 expression was increased in PC, which was also associated with poor prognosis. Furthermore, SNHG10 might be involved in the progression of PC by regulating the function of immune infiltrating cells and oxidative phosphorylation. Herein, we revealed the biological functions of SNHG10 in PC and offered a potential strategy for the diagnosis and treatment of PC.

## Data Availability Statement

The datasets presented in this study can be found in online repositories. The names of the repository/repositories and accession number(s) can be found in the article/Supplementary Material.

## Author Contributions

QC, XY, and WX collected, validated, and visualized TCGA data. QC, BG, and ZL designed and drafted the manuscript. MM, SF, and SW performed the experiments and data analysis. TS, YL, and ZZ helped to revise the manuscript. All authors reviewed and approved the published version of the manuscript.

## Conflict of Interest

The authors declare that the research was conducted in the absence of any commercial or financial relationships that could be construed as a potential conflict of interest.

## Publisher’s Note

All claims expressed in this article are solely those of the authors and do not necessarily represent those of their affiliated organizations, or those of the publisher, the editors and the reviewers. Any product that may be evaluated in this article, or claim that may be made by its manufacturer, is not guaranteed or endorsed by the publisher.

## Footnotes

1
https://portal.gdc.cancer.gov

2
https://www.ncbi.nlm.nih.gov/gds

3
http://www.gsea-msigdb.org/gsea/index.jsp

4
https://www.perl.org

5
https://cistrome.shinyapps.io/timer/

6
www.atcc.rog

## Supplementary Material

Supplementary Figure 1 The correlation between SNHG10 expression and the immune infiltration in the tumor microenvironment. (A) Differences in pDC infiltration between SNHG10 low and high expression groups. **(B)** Correlation between SNHG10 expression and pDC. **(C)** Differences in NK CD56bright infiltration levels in SNHG10 low and high expression groups. **(D)** Correlation between SNHG10 expression and NK CD56bright. NK, natural killer; pDC, plasmacytoid dendritic cell. ^∗^*P* < 0.05 and ^∗∗∗^*P* < 0.001.
