# Supplementary figures and images for: SNHG10 Is a Prognostic Biomarker Correlated With Immune Infiltrates in Prostate Cancer
Source: Front Cell Dev Biol. 2021 Oct 5;9:731042. doi: 10.3389/fcell.2021.731042 (PMC8523833; doi:10.3389/fcell.2021.731042)

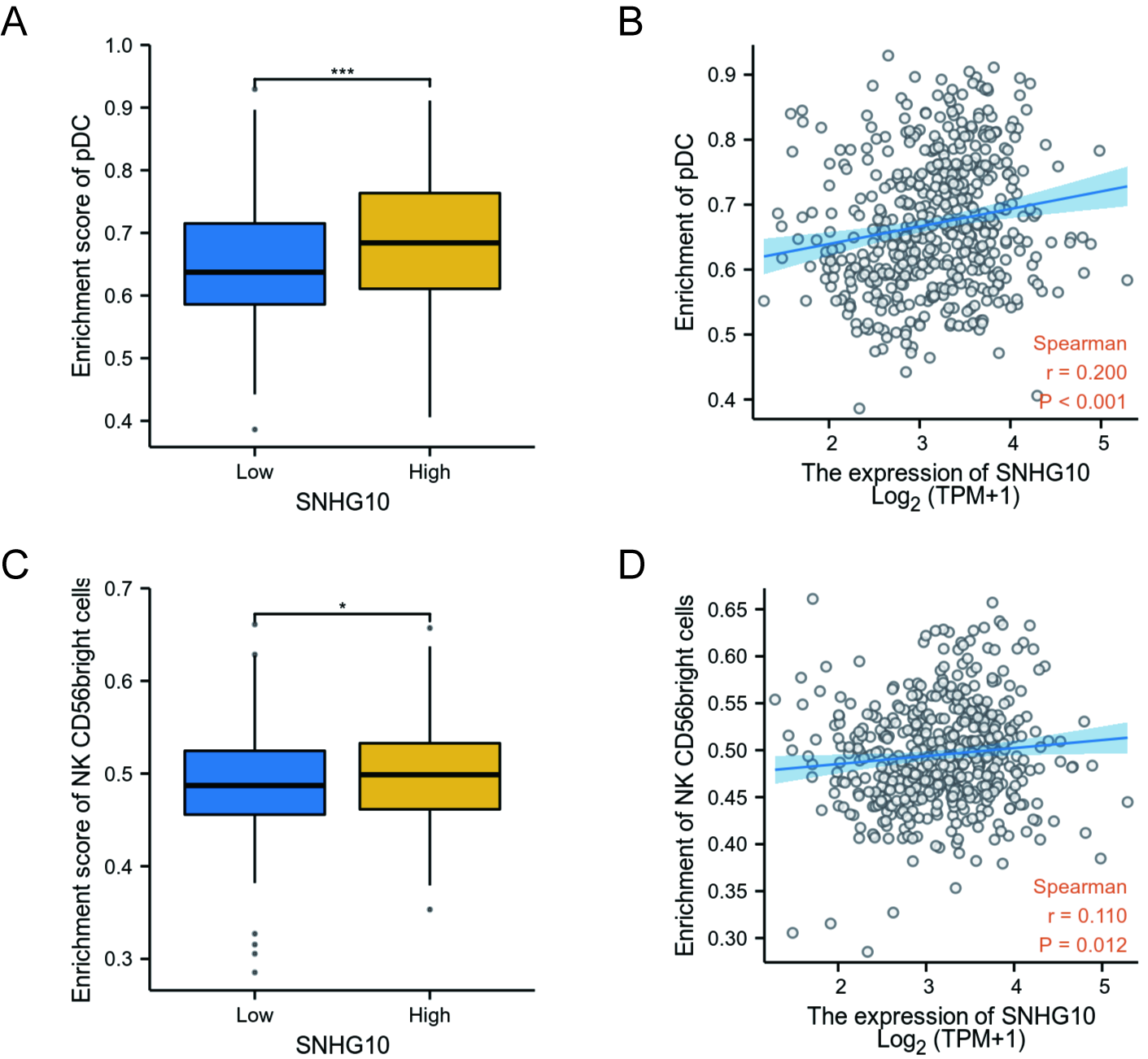

Supplement: Supplementary Figure 1 — The correlation between SNHG10 expression and the immune infiltration in the tumor microenvironment. (A) Differences in pDC infiltration between SNHG10 low and high expression groups. (B) Correlation between SNHG10 expression and pDC. (C) Differences in NK CD56bright infiltration levels in SNHG10 low and high expression groups. (D) Correlation between SNHG10 expression and NK CD56bright. NK, natural killer; pDC, plasmacytoid dendritic cell. ∗P < 0.05 and ∗∗∗P < 0.001. [file Image_1.TIF]
